# Validation of Alternative Beam T-Junction Fem Models for Complex Tubular Structures

**DOI:** 10.3390/ma15186468

**Published:** 2022-09-17

**Authors:** Francisco Badea, JoseLuis Olazagoitia, JesusAngel Perez

**Affiliations:** 1 Department of Industrial Engineering and Automotive, Nebrija University, Santa Cruz de Marcenado 27, 28015 Madrid, Spain; 2 Department of Construction and Manufacturing Engineering, Mechanical Engineering Area, University of Oviedo, 33203 Gijón, Spain

**Keywords:** finite element analysis, structural optimization, beam T-junctions, beam model validation

## Abstract

The finite element analysis of tubular structures is typically based on models constructed employing beam-type elements. This modeling technique provides a quick and computationally efficient option for calculation. Nevertheless, it shows a series of limitations related to the simplicity of this type of element, among which the inability of accounting for the stiffness behavior at the joint level is of notable importance when modeling complex tubular structures. Despite these limitations, the alternative of simulating complex tubular structures with shell- or volume-type elements is highly costly due to the complexity of the modeling process and the computational requirements. Previous research has proposed alternative beam models that improve the estimations when modeling these structures. These research validations were limited to simple models. This paper presents a validation process utilizing a previously developed beam T-junction model in a complex tubular structure, intended to be representative for buses’ and coaches’ upper structures. Results obtained reveal that the accuracy of beam element type models can be significantly improved with the adequate implementation of elastic elements to account for the real junction stiffness.

## 1. Introduction

The study and analysis of structures using the finite element method (FEM) is key to the development of complex structural systems [1]. In fact, CAD design and FEM analysis allow for the quick evolution of a sketch from a design to a structure capable of withstanding the required stresses, and these methods are widely accepted in the industry.

In FEM programs, it is possible, depending on the model to be represented, to choose three main types of elements: beam, shell, and volume elements. Although there are certain rules, the selection of the best type of element for a particular application is a generally complicated process, in which several factors must be considered. For example, it is necessary to know the characteristics and complexity of the structures to be simulated, the limitations of existing computational resources, the type of simulation, and the expected accuracy of the computational results [2,3,4].

The analyst’s experience is often key in determining the best path to take in each case. In the particular case of large tubular structures such as those that can be found in buses and coaches, it is very common, for example, to use beam-type elements, due to the flexibility, limited computational need, and speed that they allow in the analysis of complex structures [5,6,7]. Models based on beam elements are comparatively simpler than those based on shell or volume types.

Although beam elements are often the preferred choice for tubular profile structures, they have some fundamental limitations due to their simple formulation. One of the main limitations is the impossibility to faithfully reproduce the localized behavior at the joint level. In these elements, the attachment with the environment is reduced to a single infinitely rigid node [8,9], which leads to estimation errors in the calculation [10].

The use of shell and volume elements in tubular element joints makes it possible to obtain more realistic joint models [1,11]. This is possible because the joint topology at the joint level can be captured by these element types with higher accuracy. In this way, it is possible to obtain accurate models that overcome the limitations of beam-type elements in this type of tubular structures. Previous research has shown that the differences in the calculation of stiffness of tubular structures modeled with beam elements versus models made with shell or volume models can vary between 5 and 45% [10,12]. The final error depends on various parameters of the tubular elements used, such as the shape of the tube cross-section and its thickness, as well as the complexity of the overall structure. The greater the complexity of the structure, the greater the difference in stiffness calculation between models made with different types of elements, since the error induced at each joint will have a deviating effect in the global response.

Tubular structural elements can be used in many applications, such as buses and coaches. The stiffness of the structure in buses uses large tubular elements and their configuration is often considerably complex. It is usual to use beam elements in their calculation since modeling with shell or volume elements is usually very time-consuming due to the complexity of the models [9]. In addition, they require a higher computational power that on many occasions does not compensate the accuracy of the obtained results.

For this reason, it would be desirable for the modeling of this type of structure to have a calculation methodology that takes advantage of the simplicity of beam-type tubular structures but improves the results through the altered modeling of the joints. There can be found several modelling proposals in the bibliography to improve ordinary beam elements accuracy. For example, B. Horton et al. [13] proposed models with modified stiffness characteristics at the adjacent elements of the joints. The same methodology was applied in [14] to account for the local stiffness modification due to crack development in beam-type structures. Additionally, hybrid models, where shell or volume element types are used for the joints, and beam elements for the rest of the beam sections, can be found with significant improvements reported [15,16]. This shell-beam or volume-beam hybrid modelling technique is still time-consuming since connection elements are to be configured at zones where the element type is modified, and thus not attractive for larger models with an important number of joints to be configured. Finally, several approaches can be found that focus on modifying the stiffness characteristics of the joint by introducing elastic elements [8,17].

This article presents a complete methodology for comparing different modelling techniques of beam-type structures. In the study, volume, ordinary beam, and alternate beam modelling techniques, presented in [8], are analyzed. The results are validated against a real structure with respect to which the results are compared and the best methodology to obtain a model based on beam-type joints with improved T-junctions is proposed.

**Figure 1:**
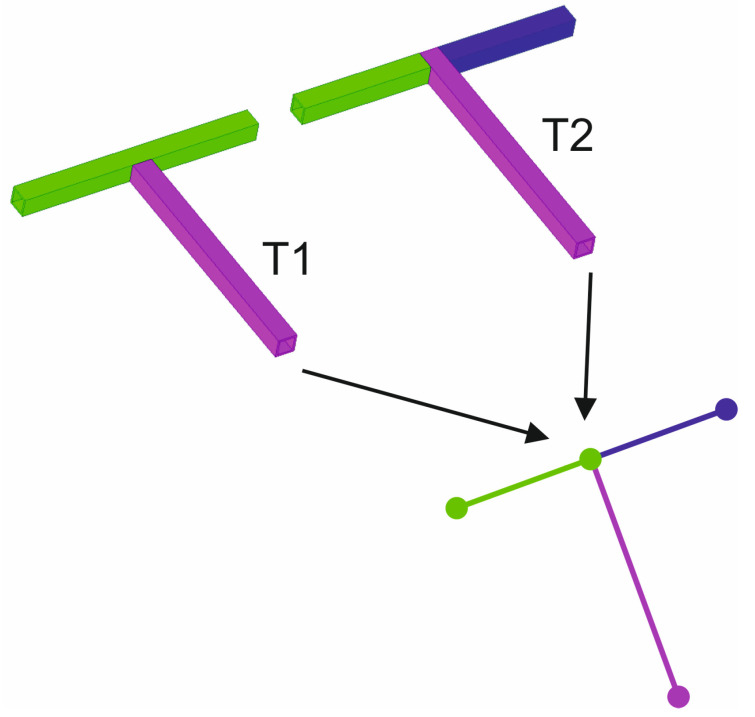
T-junctions equivalent beam-type element model. T1 junction with two elements (green and purple) and T2 with three elements (green, purple and dark blue).

To understand the methodology used, it is important to present the way of modeling the junctions between tubular elements. For simplicity, two possibilities of T-junctions between tubular elements are presented, which will be called T1 and T2. Figure 1 presents graphically the effective connections between these tubular elements.

It is important to note that the type of actual T-joint (either T1 or T2) has a direct influence on the final behavior of the structure, determining its behavior as a function of the type, size, and direction of the load applied to it. Regardless of this reality, the pure equivalent model based on beam elements will in all cases be composed of four nodes and three beam elements. Therefore, these results in the T1 or T2 junctions are blurred in the model, resulting in an infinitely stiff joint in all cases and a loss of the starting information.

**Figure 2:**
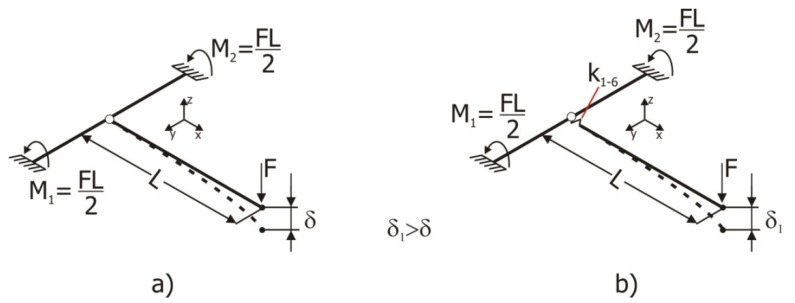
Diagrams comparing the behavior of a standard beam T-junction and the alternative beam T-junction model for the same load state. (**a**) Rigid junction (**b**) flexible junction.

To illustrate how the T-joint model of Figure 1 is modified in [8], Figure 2 presents two joint schemas, where the junction of both tubular elements is rigid in its left image (a) and is flexible in the right image (b). This flexibility is represented by the introduction of stiffness k1 to k6 at the intersection point of both tubular elements. These stiffness values are experimentally pre-determined to faithfully represent real T-joints. These stiffness values do not change the dimensions of the structure in any way. The direct effect of them into the joint causes the displacement δ1 > δ in all cases.

The consequence of the implementation of the junction-specific stiffness is an improvement in the accuracy, as it was already shown and validated for simple structural components in [8]. Nevertheless, a more realistic validation against a complex structure was still pending. This paper covers this aspect, showing the capability of the proposed methodology to increase beam-based FEM models’ accuracy and describing some key aspects to take into account when scaling from single-joint to complete complex structures. The application scope of this methodology is limited to the elastic deformation range of the structure, since the spring elements introduced are linear, i.e., k1 to k6 stiffnesses are constant. Equivalent methodology could be developed with nonlinear springs and damping elements to analyze structures responses beyond the elastic range.

In summary, the present work, applied together with the results supplied in [8], provides a complete methodology for improving beam-type element models accuracy for complex structures typically used in buses and coaches, without incurring increased computational costs and model preparation times.

## 2. Methodology

The results of the alternative T-junction model were verified for the simple T-junctions analyzed [8]. In that work, the complete methodology to obtain the proper spring stiffness values for each type of junction is described. Nonetheless, extrapolating the results to more complex tubular structures require a specific experimental validation process to assess the improvement capability of the alternative beam model proposed when crossed influences take place among the joints of the structure.

**Figure 3:**
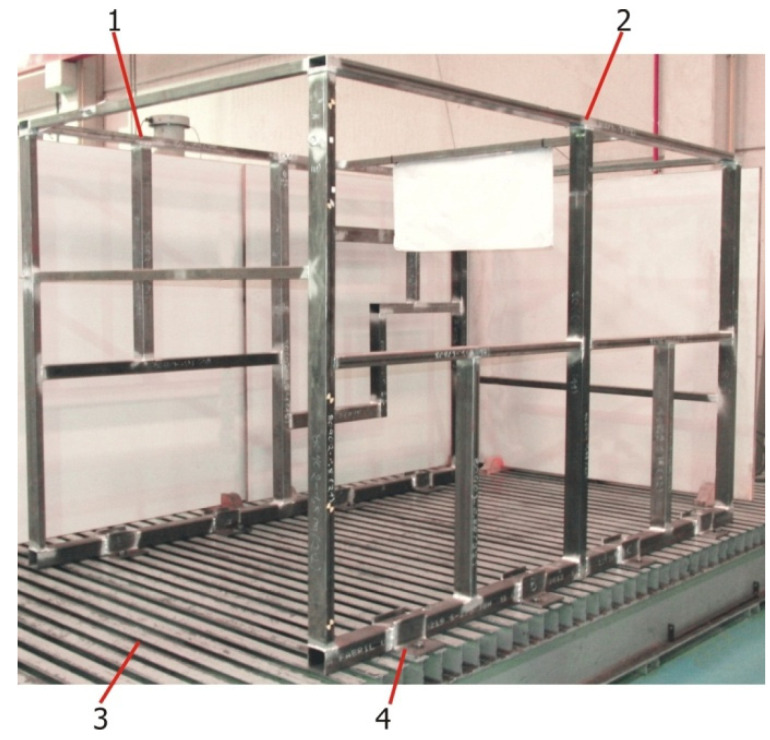
Tubular structure for the experimental validation.

As the research carried out was intended to be applied to buses’ and coaches’ upper structures, it was sought to use structures of these characteristics for the validation experiments. Since these structures are hard to come upon, a validation structure was designed and built having representative characteristics of buses’ and coaches’ upper structures (Figure 3).

The response of the validation structure under the prescribed load is captured by means of analog dials and later compared to the following finite element models:

- Model constructed with volume-type elements: The most complex and accurate modeling element type. Although unattractive for practical use in the industry, it was used as a comparison basis.
- Ordinary beam element type model: used to evaluate the improvement of the alternative beam models.
- Alternative beam element type: As will be shown in the next section, the assignment of the joint type (T1 or T2) might not be obvious in some specific cases. To analyze the influence of choosing between different criteria, three models are presented and evaluated in the paper.

A further description on the validation structure and the finite element models is given in the following section.

The measured displacements at the evaluation points are then compared to the predictions of the different models in order to assess the accuracy of the models with respect to reality and the improvements achieved with the alternative beam models. Results will be discussed in Section 4.

## 3. Experimental Validation

### 3.1. Validation Structure Description

**Figure 4:**
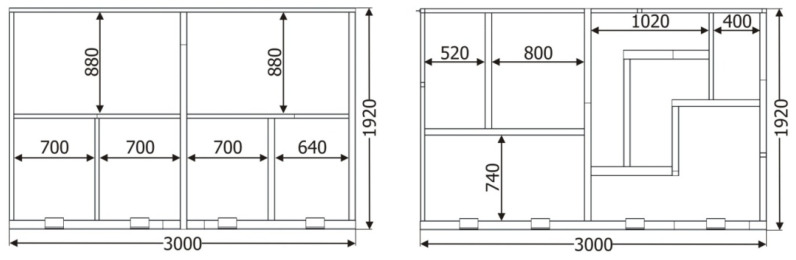
Geometric configuration of the sides of the tubular validation structure. Unit: mm.

The tubular validation structure was built with two significantly different sides from a geometric point of view. One of the sides was configured in a very similar way to what is commonly found in buses and coaches (Figure 3, number 2), whereas the opposite side had a significantly asymmetric configuration (Figure 3, number 1), so that a wide range of joint configurations is analyzed during the tests. Figure 4 shows a detail of the dimensions of both sides of the structure.

**Figure 5:**
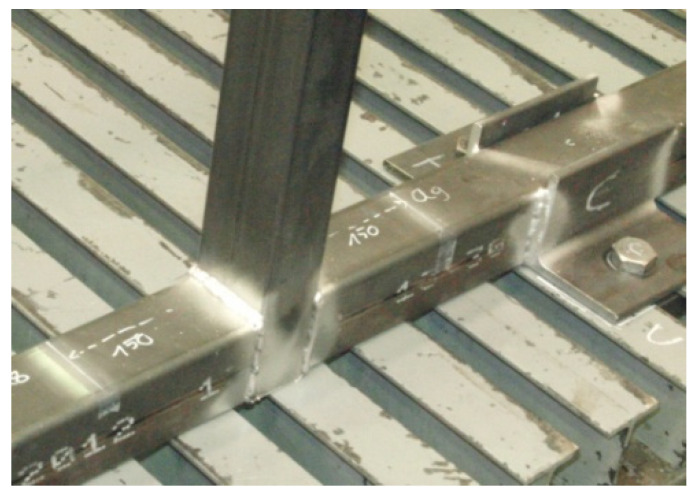
Clamping weld details.

The experiments with the tubular validation structure were done on a universal test bench (Figure 3, number 3). Additionally, a solution had to be found to properly constrain the structure during testing to avoid errors introduced by the displacement of the restriction points, since these displacements are not accounted for on virtual FEM models. To minimize these errors, 100 mm × 100 mm × 10 mm L-shaped profiles welded to the base profiles of the tubular structure (Figure 3, number 4) were used. In all, 8 restriction points were used. To ensure proper clamping at the restrictions’ points, bolts were tightened to 70% of their yield strength limit. Figure 5 illustrates welds in one of the base profiles with the clamping devices and a rectangular profile.

### 3.2. Experimental Setup

**Figure 6:**
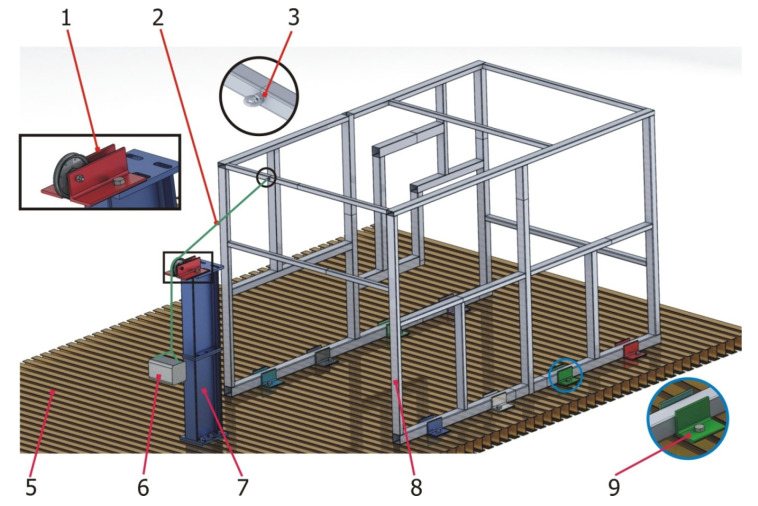
Three-dimensional CAD model with the characteristics of the experimental assembly corresponding to the testing of the validation structure.

The following Figure 6 shows an overview of the experimental setup assembly of the validation structure over the test bench. The structure (Figure 6, number 8) was conceived to be tested on a universal test bench (Figure 6, number 5) for which clamping devices had to be used (Figure 6, number 9), which were first welded to the base profiles of the validation structure and then bolted to the universal test bench with M20 bolts and threaded metal blocks.

In order to minimize uncertainties with respect to the force applied, a calibrated weight was used (Figure 6, number 6) as a load input.

With the objective of achieving a wide range characterization of loading, composed load states were input to the structure. The direction of the force was accurately selected by changing the orientation of the pulley (Figure 6, number 1) that conveys the force of the nylon cord. Additionally, by using H-shaped test bench supports, the height of the pulley could be changed to modify the vertical angle of the force applied (Figure 6, number 7). Finally, a deviation in the transverse direction was obtained by changing the position of the H-shaped test bench supports in that direction.

The load was transferred to the structure by means of a nylon thread (Figure 6, number 2) tied to the corresponding eyebolts (Figure 6, number 3).

**Figure 7:**
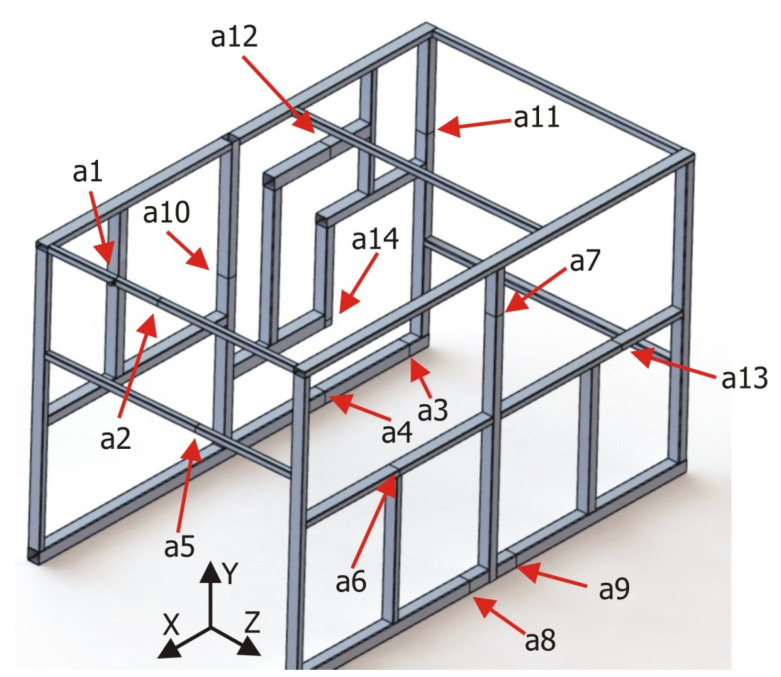
Sections used to characterize the validation structure, identified as a1 to a14.

To obtain a complete and representative characterization of the validation structure, a total of 14 measuring points were defined and distributed throughout its geometry so that representative information of the global response was obtained. It was also taken into consideration the necessity to define measuring sections at different distances from the joints themselves and/or the clamping devices, so that a clearer picture of the behavior of the beam-type elements could be studied. Figure 7 shows a 3D CAD model of the validation structure on which the defined measuring sections are indicated.

Two dial gauges were used to capture the displacement at the measuring sections, one to measure small displacements of 0–5 mm with a 0.001 mm resolution, and another for larger displacements of 0–25 mm with a 0.01 mm resolution.

Due to the number of sections and their different locations in the validation structure, specific supports were used to fix the dial gauges depending on the sections measured.

Of the 14 sections defined, in four of them (a3, a4, a8, and a9), only the displacements in the Y direction were characterized since these sections were in the base profiles of the structure in regions very close to the clamping devices, so the displacements in the other directions could be neglected. In the rest of the sections, displacements in the two most significant directions were characterized. For example, in section a1, the displacements were measured in directions X and Y, whereas in section a13, the displacements were measured in directions Z and Y.

**Figure 8:**
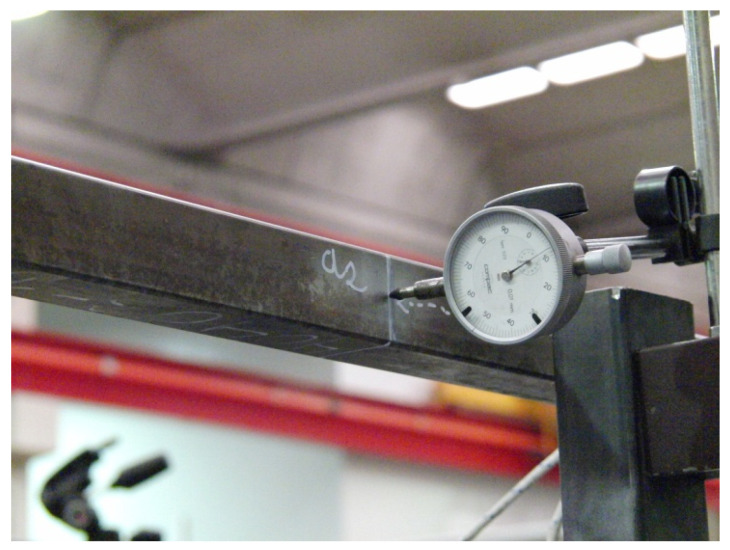
Mounting of the dial gauge for the displacement measurement of the a2 section.

**Figure 9:**
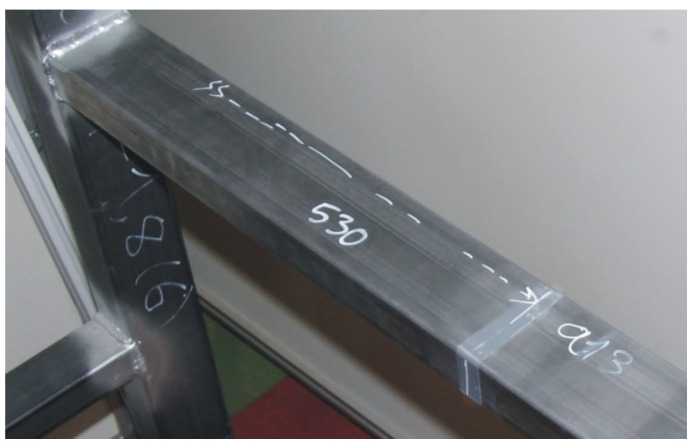
Detail of the region adjacent to the a13 measurement section.

As with the validation experiments for the simple junctions [8], the following set of criteria was adopted to minimize the sources of error and achieve better-quality results:

1. To attach the dial gauges, the back clamping system was used in order to avoid any differences in measurement due to friction between the sensor and the standardized clamping system. By way of example, Figure 8 presents the mounting of the dial gauge for the displacement measurement of the a2 section.
2. A semi-automatic system was used to read and acquire the data from the dial gauges by using a high-resolution photographic camera with an external trigger in order to avoid estimation errors between measurements.
3. The defined sections were cleaned and smoothed using solvents and scouring pads with rough polymer fibers so the surfaces would not show any defects. Adhesive strips were also attached to each of these sections. As an example, Figure 9 shows a detail of the region adjacent to the a13 measurement section.

The same preparation process was used for each of the sections defined in the validation structure. To accept the measurements, the same quality criteria were used as for the experimental analyses of the simple junctions [8]. In this way, the standard deviations for a set of measurements in the same section were less than 0.005 mm for the 0.001 mm resolution dial gauges, and less than 0.05 mm for the 0.01 mm resolution dial gauges. For each of the sections defined, load and unload cycles were carried out by means of a hydraulic actuator which gave and released support to the calibrated weights during each cycle. A total of 15 measurements were taken for each section, which were found to be an optimal compromise for obtaining precise results with the least number of measurements per point.

### 3.3. FEM Model of the Validation Structure Modeled with Beam-Type Elements

**Figure 10:**
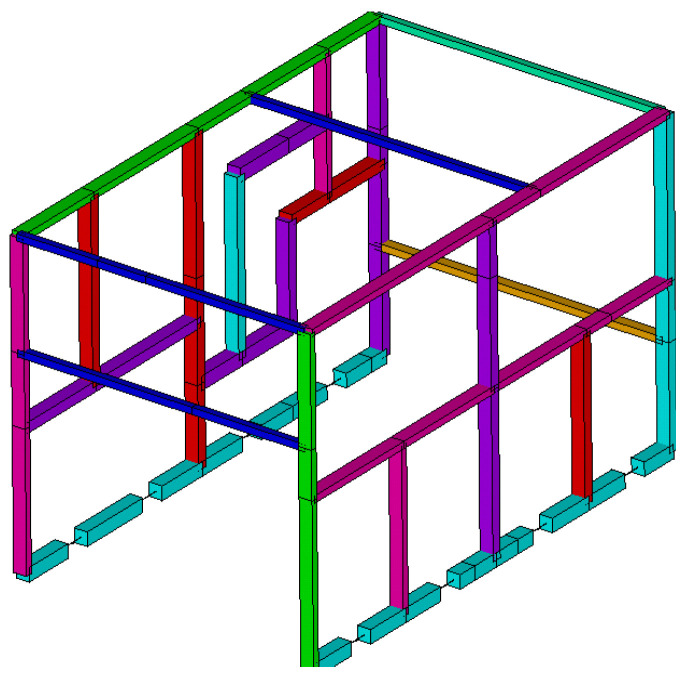
Validation structure modeled with beam-type elements. Beam colors refer to the following sections employed: Light blue: 80 mm × 80 mm × 3 mm, red: 80 mm × 60 mm × 3 mm, purple: 80 mm × 60 mm × 2 mm, green: 80 mm × 40 mm × 3 mm, lilac: 80 mm × 40 mm × 2 mm, dark blue: 40 mm × 40 mm × 3 mm.

To model the validation structure with beam-type elements, the modeling principles set out in [8] were adopted. Following the methodology proposed in this sub-section, the principal axes for each of the tubular profiles of the structure were identified and extracted. The regions where there were separations between the beam elements were also identified and corrected. Figure 10 shows the validation structure model built with beam-type elements.

This figure presents two superimposed representations of the validation structure. The original base model built with beam-type elements is represented with black dotted lines, to which the model generated by a graphic option of the program was superimposed to reveal the elements, based on the cross-section properties defined. Key points were defined at the locations of the measuring sections in order to ensure that a node would be present in these points and therefore nodal displacement results could be extracted.

**Figure 11:**
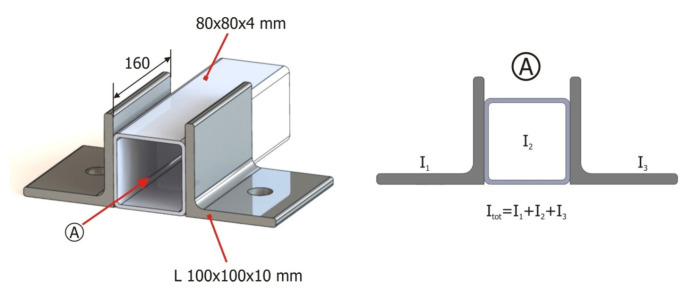
Characteristics of the structure and calculation of the moment of inertia equivalent to the cross-section in the regions of the clamping devices.

The equivalent moments of inertia were calculated in the cross-section of the clamping devices and were assigned to the beam elements, as shown in Figure 11.

### 3.4. Characteristics of the Validation Structure Modeled with Alternative Beam T-Junctions

**Figure 12:**
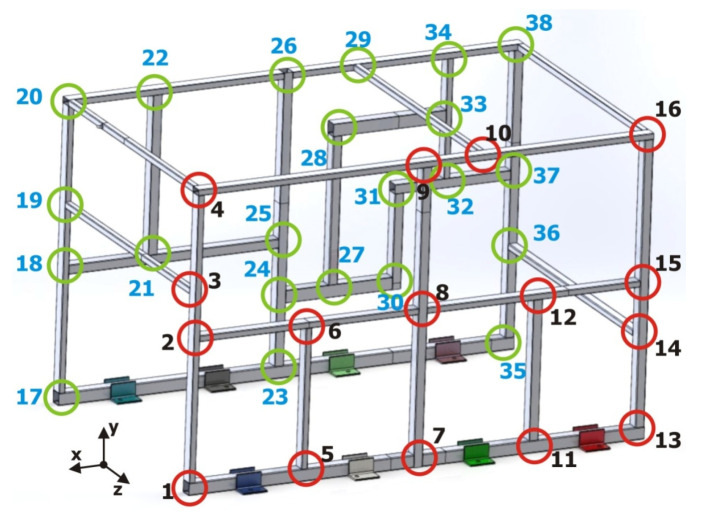
Diagram identifying the total number of joints in the validation structure.

Implementing the alternative beam T-junction elements in accordance with the developed methodology requires determining the dimensional characteristics and the configuration of each of the joints (T1 or T2). A total of 38 joints were identified in the validation structure, as can be seen in the diagram presented in Figure 12. In this figure, each one of the junctions was identified with a circle and a number; for clarity purposes, one of the sides has red circles and the other side green circles.

**Figure 13:**
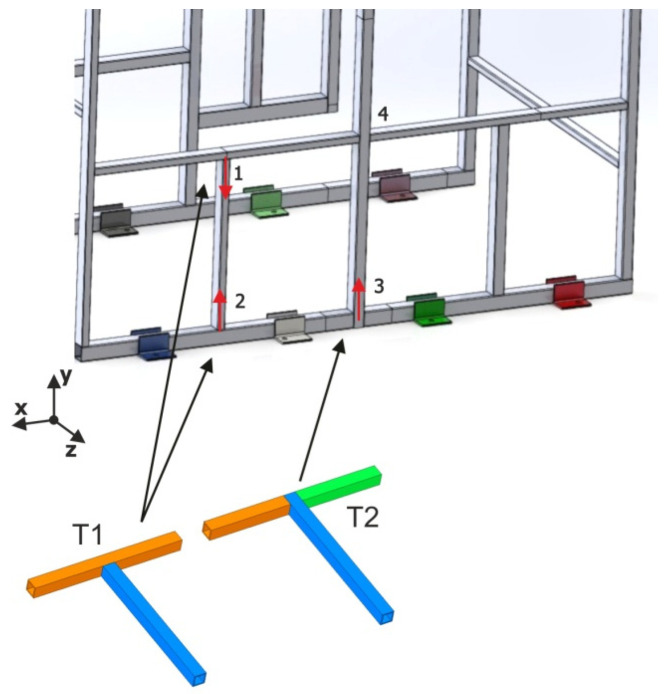
Equivalence of the complex structure joints to the T1 and T2 junctions.

Implementing the alternative junctions into a complex tubular structure requires identifying each of the junctions of the structure in accordance with the analyzed joint configurations (T1 or T2). As presented in Figure 13, most of the junctions can be easily assimilated with T1 or T2, as is the case with points 1 to 3, but some others cannot be easily determined or just have no clear equivalence. Over the 38 joints, 33 of them were easily assimilated to T1 or T2, whereas for 5 joints (4, 8, 16, 20, and 38), this similitude was not obvious.

**Figure 14:**
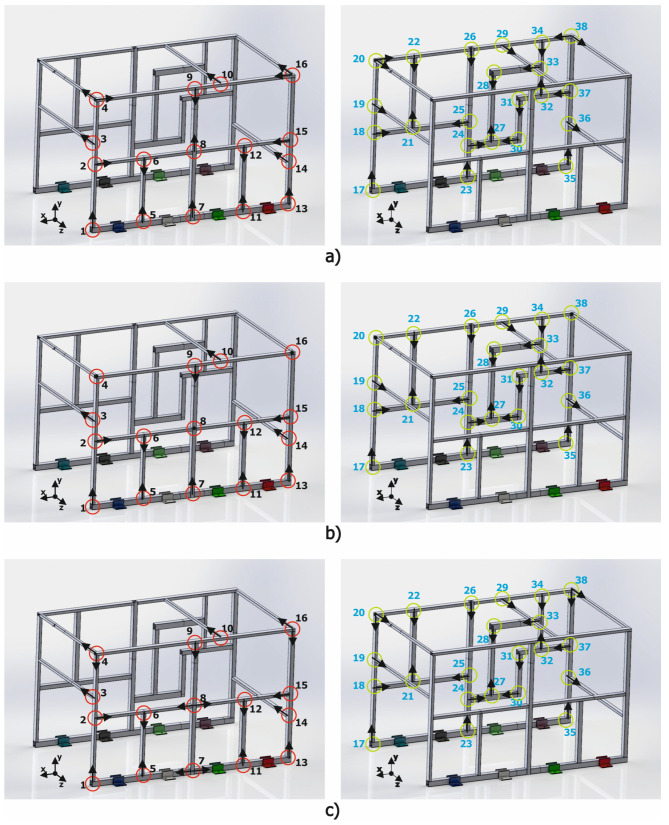
Joint configurations analyzed. Variant (**a**), Variant(**b**) and Variant (**c**).

To further analyze the junctions with no clear equivalence to T1 or T2 configurations, different models with various combinations were developed and evaluated. Although a complete description of all the combinations analyzed is out of the scope of the article, the best three configurations will be described in the following paragraphs (Figure 14).

For the first variant (a), joint 8 was modeled as a single type T2 joint, whereas for joints 4, 16, 20, and 38, the modeling characteristics were adhered to (a T1 and a T2) but changed the direction of application of the type T2 junction. For the second variant (b), it was decided to model all the type T1 and T2 joints that could be clearly identified in the structure and to keep all the joints showing uncertainties (8, 4, 16, 20, 38) as normal junctions. Finally, for the third variant (c), all the conflictive joints were assimilated as type T1 joints, keeping all the continuous profiles unmodified.

The stiffness values of the elastic elements used for each of the analyzed joints were found through comparative simulations using joints modeled with volume-type elements as reference models, following the recommendations of [8].

It should be highlighted that the stiffness values at the joint levels obtained with the methodology of [8] are significantly high, ranging between 1 × 10^5^–1 × 10^6^ (N/m). These stiffnesses in some manner quantify the contribution of the joint to the deformation of the T-junctions in the linear domain. Ideally, it would be desired to improve each junction with sets of elastic elements so that the complex beam structure provides the most accurate possible results. Despite that the assimilation to a joint type in 5 out of the 38 joints of the validation structure is not obvious, it was demonstrated in [8] that although between the T1 and T2 junctions there are significant differences, these differences were comparatively lower than the ones in comparison to the regular beam junction. In other words, assigning a junction as T1 when it is a T2 would induce fewer deviations than just having it as a regular beam.

### 3.5. Characteristics of the Validation Structure Modeled with Volume-Type Elements

An additional model of the validation structure was constructed using volume-type elements. Although modeling rectangular beam section structures with volume-type elements has been demonstrated to be an excessively costly approach, it was decided to configure this model to be used for comparison purposes, due to its intrinsic high accuracy.

**Figure 15:**
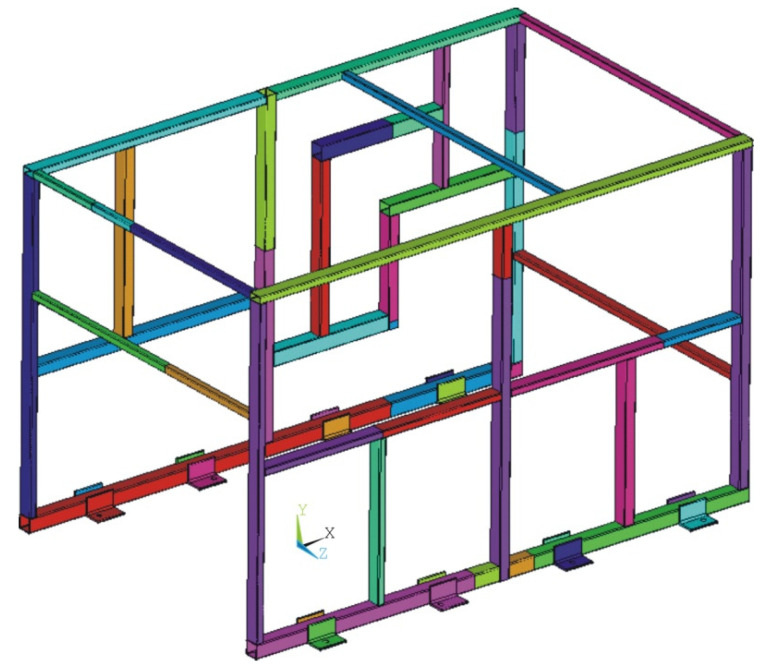
Detailed model of the validation structure, modeled with volume-type elements.

The following Figure 15 illustrates the structure modelled with volume-type elements. It was modelled using linear hexahedral elements of size 3 mm, which corresponds to the minimum thickness of the rectangular beams employed in the validation structure. Tube joints were modeled by means of bonded-type contacts, without considering the weld seam geometry.

## 4. Results and Discussion

Comparative analyses were performed for the evaluation of the improvements between the validation structure and the different finite element models of the validation structure. Table 1 summarizes the displacement relative deviations for each of the finite element models evaluated with respect to the values measured in the experimentally analyzed validation structures.

From the observation of the results Table 1, the following aspects can be observed:

1. The best approximations obtained correspond to the detailed volume-type element model.
2. The beam and the alternative beam models show significant deviations for sections a3, a4, a8, and a9 (Figure 7). These sections are located at short distances from the clamps, for which local effects are influencing the results, which cannot be characterized using beam-type elements. It can also be seen that these sections do not undergo any significant changes in the alternative beam models.
3. Focusing on alternative beam models, variant (c) shows the best approximations to the experimental validation structure and to the detailed volume element model. The calculations are even better displacement estimations than those of the latter at a5 and a10 sections.

Since the ultimate objective of the implementation of alternative beam models is to improve the accuracy of tubular structures modeled in this manner, it was considered necessary to perform an additional evaluation of the capability of the models to predict stress distributions.

It was observed in [8] that the stress distributions resulting from analyzing the simple junctions modeled with beam or alternative beam type elements showed no significant differences. Nevertheless, it seems interesting to extend the analysis to complex structures, where the effect of the local stiffness modification at the joint level might show crossed influences.

Due to the inherent limitation of beam-type elements to account for local stress raisins at the joint level, the analysis is focused on the comparison of the general stress distribution maps. The volume element type model was used as a reference for the evaluation, since this type of model, although discarded for general use due to the high computational costs that it entails, can predict stress maps with high precision.

**Figure 16:**
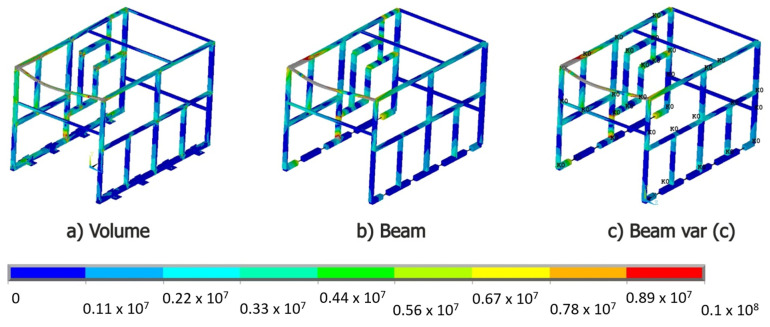
Von Mises stress maps for the validation structure modeled with volume, beam, and alternative beam type elements [MPa].

The resulting Von Mises stress distributions obtained for volume, ordinary beam, and the alternative beam with the best results (c) are showed in Figure 16. It is observed that both ordinary and alternative beam models show rather similar stress distributions, which are also similar to the reference volume model.

Although a detailed stress analysis of the models is not in the scope of the article, it is noted that the significant improvement in terms of deflection observed on the alternative beam model has limited influence on the general stress distribution for complex structures.

## 5. Conclusions

In the present paper, a test of specific complex structures was designed and built in order to conduct the validation of the alternative beam element type modeling technique presented in [8].

The structure was subjected to a controlled load, and displacements were captured at a total of 14 measuring sections for comparison purposes.

For such complex structures, the application of the stiffness accuracy improvement by means of elastic elements becomes less obvious, since the assignation of T1 or T2 joints’ configuration is not evident anymore for some joints. In the case of the validation of the structure used in this work, a total of five junctions were identified in which the selection was not clear and so the optimization of those particular junctions implied a detailed specific analysis for which different configurations were modelled for these junctions and compared to the experimental data, together with an ordinary beam element type and a volume element type model.

For the ordinary-type elements, the deviations with respect to the validation structure were found to be notable, with the beam model being approximately 58% stiffer.

By using the developed alternative T-junctions, it was shown that the characteristics of beam models’ behavior can be modified. Evaluating the differences between the experimental structure and the models built, and ignoring the measurements corresponding to the support beams of the structure (sections a3, a4, a8, and a9), a reduction in the average deviations from almost 49% to 14% was achieved. It was also noticed that these models are indeed affected by the proper choice of elastic element configuration, ranging from an average error of 27% to 14%. In any case, even the worst selection of the elastic elements for the junctions shows a significant accuracy improvement with respect to the ordinary beam model. Additionally, by analyzing the stress distributions in the different finite element models, it was found that the elastic elements inserted into the joints did not show significant influence on the stress distribution.

From the results presented in this work, it can be concluded that the utilization of the alternative beam T-junction model for the behavioral optimization of tubular structures represents a feasible methodology throughout which significant improvements of the analyzed model estimations can be obtained.

The authors would like to remark that, when performing studies throughout finite element analysis, it is necessary to take into consideration the fact that the beam-type elements represent a simplified element derived from the shell- and volume-type elements, which implies a series of intrinsic limitations determined by their own formulation.

## Figures and Tables

**Table 1:** Deflection relative differences of the FEM models with respect to the experimental results.

	Exp Volume Deviation	Exp Beam Deviation	Exp Alt Beam Var (a) Deviation	Exp Alt Beam Var (b) Deviation	Exp Alt Beam Var (c) Deviation
Characterized sections	[%]	[%]	[%]	[%]	[%]
**a1**	6.03	43.96	15.80	33.21	13.89
**a2**	5.93	43.39	15.91	33.87	15.57
**a3**	15.38	67.35	66.88	65.63	60.54
**a4**	8.43	97.35	97.34	97.21	97.29
**a5**	13.46	45.58	13.14	23.15	11.20
**a6**	6.01	88.95	69.83	64.87	23.54
**a7**	6.59	35.49	15.43	12.98	9.31
**a8**	44.64	71.09	81.14	82.46	82.24
**a9**	21.55	94.57	89.25	82.22	82.25
**a10**	6.22	40.25	1.16	3.30	2.87
**a11**	6.68	32.40	15.58	10.73	8.54
**a12**	12.37	49.23	14.25	13.16	14.21
**a13**	17.41	56.96	26.81	22.89	23.50
**a14**	14.46	53.30	48.95	27.48	21.28
**Absolute average deviation**	13.23	58.56	40.81	40.94	33.30
**Absolute average deviation** **without clamps (discarding the a3, a4, a8, a9 values)**	9.52	48.95	23.69	24.56	14.39

## Data Availability

Not applicable.
